# A Novel Model for Student-Led Justice, Equity, Diversity and Inclusion Training at a UK Medical School

**DOI:** 10.1192/bjo.2024.296

**Published:** 2024-08-01

**Authors:** Isabelle Gallier-Birt, Emily Róisín Reid, George Webster, Mary Bowley

**Affiliations:** University of Warwick, Coventry, United Kingdom

## Abstract

**Aims:**

It was identified that at Warwick Medical School (WMS) there was no provision for in-person, student-led Justice, Equity, Diversity, and Inclusion (J, E, D&I) training for both staff and students. A novel approach using case-based studies and group discussions was developed through a student-staff collaboration with the aim of participants gaining a greater understanding of the impact of various institutional practices, from the perspective of students with first-hand experience of the subject matter. The training aimed to promote a greater understanding of intersectionality, and how institutional practices can disproportionately disadvantage students depending on their identity, experience, and background. Participants were encouraged to reflect upon the cumulative effects of systemic disadvantages in higher and medical education. The subsequent impact upon academic attainment, mental health and wellbeing was a further focus.

**Methods:**

Around 350 students and staff from across WMS attended the training sessions over 6 months. These sessions were led by a team of student facilitators who possessed subject expertise in topics related to J, E, D&I, in addition to representing the communities that were discussed in terms of inclusion. The content was delivered in the form of case-based scenarios and small and wider group discussions. Content was based on discussions surrounding racism, classism, ableism, homophobia, sexism, Islamophobia, and transphobia. Discussion was encouraged and facilitated to promote reflection on personal practices and acknowledgement of where future efforts to improve practice should be directed.

**Results:**

Results indicate statistically significant shifts in participant knowledge and confidence levels in pre-post survey data, with qualitative feedback emphasising the strength of the student-led format. Faculty and students commented on the benefit of student-lead case-based teaching and student facilitator reflections highlight personal growth and the challenges of navigating power dynamics.

**Conclusion:**

Overall, this project illustrates the efficacy of a student-led change initiative in fostering inclusivity and positive change within educational environments and provides an original model to explore for future partnership-working across the medical school. The student-led approach facilitated mutual learning between staff and students, bringing greater focus to how student attainment and wellbeing can be impacted by institutional practices.

